# 

**Published:** 1863-07

**Authors:** 


### CONTENTS.

ORIGINAL ESSAYS AND COMMUNICATIONS.

Development of Dental Tissues. By W. H. A ........ 279
An Address, by Dr. S. B. Palmer.. ........ 285
To Remove Amalgams and Re-fill with Gold........ 290

PROCEEDINGS OF SOCIETIES.

Meeting of Dental Society of Western New York........ 293

SELECTIONS.

Alveolar Abscess........ 299
Animal Life........ 304
Scavengers of Nature........ 306
Restoring to its Natural Appearance a Putrified Dead Body........ 308
American Dental Convention........ 314
Suggestion........ 315

EDITORIAL.

Case, No. 11 and 12........ 316
American Dental Convention........ 318
American Dental Association,........ 318
Trip East........ 319
Odontographic Society of Pennsylvania........ 319
Something New........ 319
American Hard Rubber Co. against John T. Toland........ 319

